# Neonatal idiopathic primary hypoparathyroidism: A rare cause of neonatal seizures

**DOI:** 10.12669/pjms.315.7807

**Published:** 2015

**Authors:** Shabbir Hussain, Moin-ud-Din Sabir, Mubaral Ali, Syed Awais-ul-Hassan Shah

**Affiliations:** 1 Shabbir Hussain, MBBS, FCPS, Clinical fellowship in neonatology (UK). Consultant Paediatrician/Neonatologist, Combined Military Hospital, Peshawar, Pakistan; 2 Moin-Ud-Din Sabir, MBBS, Combined Military Hospital, Mardan, Pakistan; 3 Mubaral Ali, MBBS, Combined Military Hospital, Peshawar, Pakistan; 4 Syed Awais-ul-Hassan Shah, MBBS, Combined Military Hospital, Peshawar, Pakistan

**Keywords:** Neonate, seizure, Hypocalcaemia, Hypoparathyroidism, Hyperphosphatemia

## Abstract

Etiology of neonatal seizures (NNS) is diverse and hypocalcemia is one of the treatable causes. Neonatal hypocalcemia (NHC) due to congenital hypoparathyroidism, either permanent or transient, is extremely rare. Its biochemical abnormalities include hypocalcemia, hyperphosphatemia and low levels of intact parathyroid hormone (PTH). Isolated congenital hypoparathyroidism in which deficiency of PTH has no association with maternal, syndromic or endocrine defects is a very rare entity. We are reporting a case of a newborn who presented with seizures on the 5th day of life and later on investigations revealed hypocalcaemia due to isolated congenital hypoparathyroidism.

## INTRODUCTION

Seizures in the neonatal period have diverse etiology and hypocalcaemia is one of the treatable metabolic causes.^1^,^2^ Neonatal hypocalcemia may be transient or permanent, early or late onset and primary or secondary. Hypoparathyroidim is one of the rare causes of late onset neonatal hypocalcemia. Isolated congenital (idiopathic) primary hypoparathyroidism is a rare congenital entity which is usually sporadic but familial cases have also been reported.^2^,^3^ Hypoparathyroidism can also occur as a part of maternal, autoimmune disorders, polyendocrinopathy, with dysmorphic syndromes like CATCH 22, DiGeorge, velo-cardiofacial defect, post thyroidectomy complications and basal ganglia calcification.^2^,^4^-^6^ True incidence of hypoparathyroidism is not known but from Japan it is reported as 7.2/million people.

## CASE REPORT

A male neonate presented on 18^th^ day of life with history of recurrent seizures for the last 14 days. Seizures were focal and characterized by twitching of eyes, jerky movements of limbs, multiple episodes per day, brief in duration, about 1-2 minutes and seized spontaneously. The baby remained well between the seizures. There were no associated features like fever or reluctance to feed. He was born as spontaneous vertex delivery in a hospital to consanguineous parents as twin pregnancy. The other twin remained healthy and seizure free. He had one elder female sibling who was alive and healthy. No history of maternal illness or drug intake during pregnancy and antenatal records were unremarkable.

On examination he was irritable, Vital signs were within normal limits, anthropometric data at 50^th^ centile, systemic examination unremarkable and no dysmorphic features. Focal seizures were observed characterized by twitching of eyes, jerky movements of limbs and were controlled with I/V Midazolam. Baseline investigations were sent along with a partial septic screening. Broad spectrum antibiotics were started along with supportive paraphernalia.

Results of investigations revealed normal blood counts, Renal function tests, blood glucose, electrolytes and C reactive protein but hypocalcemia was noted. Intravenous calcium replacement was done and seizure stopped. He remained well for 24 hours and again started having seizures. Again metabolic screening (blood glucose, calcium, magnesium, electrolytes) Inborn error of metabolism screening (ABGs, lactate and ammonia) and Ultrasonography brain were done. Again it revealed hypocalcemia. To find out the cause of persistant hypocalcemia, further laboratory workup (phosphate, alkaline phosphatase, vit D and PTH levels) was done. It was suggestive of hypoparathyroidism. X-Ray chest and Echocardiography were done to rule out DieGorge syndrome. ECG revealed prolonged Q-T interval. Maternal bone profile and PTH level were within normal limits. A final diagnosis of congenital primary hypoparathyroidism was made and patient managed with I/V calcium and oral cholecalciferol. Seizures seized completely and on 7^th^ day of hospitalization patient was discharged on oral medication (calcium and cholecalciferol) with follow up advice. At 6 month follow up he is thriving well with no evidence of nephrocalcinosis or any other complication and metabolic profile is within normal range.

## DISCUSSION

Hypocalcaemia is a common metabolic problem of neonatal period. Neonatal hypocalcemia is defined as total serum calcium of less than 7 mg/dL (1.75 mmol/L) or ionized calcium less than 4 mg/dL(1 mmol/L) in preterm neonates and less than 8 mg/dL (2 mmol/L; total) or <1.2 mmol/L (ionic fraction) in term neonates.^7^ It is divided into early and late onset depending upon age of neonate. Late onset hypocalcaemia usually occurs at the end of first week of life and hypoparathyroidism is a rare cause of late onset hypocalcaemia.

Hypoparathyroidism can occur in isolation or in combination with other autoimmune/genetic defects.^2^,^4^-^6^ Sanjad and Sakati have described such associations of hypoparathyroidism with facial dysmorphism, growth failure and mental retardation.^8^ DiGeorge syndrome has also association with hypocalcemia. Isolated cong hypoparathyroidism can occur as a sporadic or familial disorder with inheritance by autosomal dominant, recessive or X linked modes of transmission.^9^ Defects in the prepro PTH gene located at 11p15 gene locus and mutations in calcium sensing receptor gene (3q21-24) have been associated in cases of isolated PTH deficiency.^10^

One of the differential diagnosis is hypomagnesemia. It can lead to decreased levels of calcium, Vit D and PTH. But if we correct hypomagnecemia with parenteral magnesium then calcium, vit D and PTH levels are automatically corrected.

**Table:** Investigation Summary

Serial	Investigation	Result	Values
1:	CBC	Normal
2:	CRP	< 6mgm/dl (Normal)
3:	GLUCOSE	60 mg/dl
4:	AMMONIA	40 umol/l	12-60umol/l
5:	LACTATE	0.8mmol/l	0.5-2.2 m mol /l
6:	ABGs	Normal
7:	USG BRAIN	Normal study
8:	XRC	Normal
9:	ECHOCARDIOGRAPHY	Normal
10:	ECG	QT c-prolonged
11:	MAGNESIUM	0.9mmol/l	0.7-1.1mmol/l
12:	CALCIUM	1.5mmol/l	2.1-2.6mmol/l
13:	PHOSPHATE	6.5mg/dl	2.5-4.5mg/dl
14:	VIT D	15ng/ml	20-150ng/ml
15:	PTH	9pg/ml	14-67pg/ml

In our case patient presented on the 5^th^ day of life with seizures and had hypocalcemia, raised phosphate, normal magnesium and low PTH levels in comparison with two cases presented by Atika^2^ of isolated congenital hypoparathyroidism. Patient was treated with calcium supplementation and remained symptom free till 6^th^ month of life, after that the follow up was lost. Similarly Rocha et al presented a case with hypocalcaemia and low PTH levels but in contrast to ours their patient had hypomagnesaemia.^11^

Aim of reporting this case is to emphasize that at times, presentation of extremely rare disorders may mimic with very common illnesses but a high index of suspicion should be kept in mind to diagnose rare disorders like neonatal congenital primary hypoparathyroidism.
